# A Comparison of Molecular Biology Mechanism of *Shewanella putrefaciens* between Fresh and Terrestrial Sewage Wastewater

**DOI:** 10.3389/fbioe.2016.00086

**Published:** 2016-11-04

**Authors:** Jiajie Xu, Weina He, Zhonghua Wang, Dijun Zhang, Jing Sun, Jun Zhou, Yanyan Li, Xiurong Su

**Affiliations:** ^1^School of Marine Science, Ningbo University, Ningbo, China; ^2^College of Engineering, China Agricultural University, Beijing, China; ^3^Department of Food Science, Cornell University, Ithaca, NY, USA

**Keywords:** *Shewanella putrefaciens*, differential proteomics, metabolomics, real-time quantitative PCR

## Abstract

Municipal and industrial wastewater is often discharged into the environment without appropriate treatment, especially in developing countries. As a result, many rivers and oceans are contaminated. It is urgent to control and administer treatments to these contaminated rivers and oceans. However, most mechanisms of bacterial colonization in contaminated rivers and oceans were unknown, especially in sewage outlets. We found *Shewanella putrefaciens* to be the primary bacteria in the terrestrial sewage wastewater outlets around Ningbo City, China. Therefore, in this study, we applied a combination of differential proteomics, metabolomics, and real-time fluorescent quantitative PCR techniques to identify bacteria intracellular metabolites. We found *S. putrefaciens* had 12 different proteins differentially expressed in freshwater culture than when grown in wastewater, referring to the formation of biological membranes (Omp35, OmpW), energy metabolism (SOD, deoxyribose-phosphate pyrophosphokinase), fatty acid metabolism (beta-ketoacyl synthase), secondary metabolism, TCA cycle, lysine degradation (2-oxoglutarate reductase), and propionic acid metabolism (succinyl coenzyme A synthetase). The sequences of these 12 differentially expressed proteins were aligned with sequences downloaded from NCBI. There are also 27 differentially concentrated metabolites detected by NMR, including alcohols (ethanol, isopropanol), amines (dimethylamine, ethanolamine), amino acids (alanine, leucine), amine compounds (bilinerurine), nucleic acid compounds (nucleosides, inosines), and organic acids (formate, acetate). Formate and ethanolamine show significant difference between the two environments and are possibly involved in energy metabolism, glycerophospholipid and ether lipids metabolism to provide energy supply, and material basis for engraftment in sewage. Because understanding *S. putrefaciens*’s biological mechanism of colonization (protein, gene express, and metabolites) in terrestrial sewage outlets is so important to administering and improving contaminated river and to predicting and steering performance, we delved into the biological mechanism that sheds light on the effect of environmental conditions on metabolic pathways.

## Introduction

With the increase of human activities and industrial processes, more urban and industrial wastewaters are discharged to rivers or oceans without appropriate treatment, especially in developing countries (Yan et al., 2015). In the last decade, marine pollutions were exposed to a variety of chemical compounds (Benedetti et al., 2015; Fabbri, 2015), leading to ocean environmental pollution and enormous biodiversity disease outbreaks, which can damage further human health (Harvell et al., 1999; Solaun et al., 2013; Prest et al., 2014). The land-based pollution is recognized as one of most serious marine pollution sources worldwide contributing to more than 75% of the pollutants entering the sea (Zhao et al., 2015). Hence, it is urgent to control and treat the contaminated river.

*Shewanella putrefaciens* belongs to the genus *Shewanella*, family Shewanellaceae, order Alteromonadales, class γ-Proteobacteria, and phylum Proteobacteria. The bacterium is a Gram-negative non-fermentative oxidative bacillus, which was previously known as *Pseudomonas putrefaciens*, first isolated by Debby and Hammer (1931). It is commonly found in water-related environments such as freshwater, seawater, lakes, rivers, sewage, fish, and soil (Bulut et al., 2004; Basir et al., 2012). *S. putrefaciens*, a specific spoilage bacterium of iced fresh fish regardless of the origin of the fish (Gram and Huss, 1996) has an extensive growth range and ability to survive in harsh environments. Under static and low oxygen conditions, *S. putrefaciens* isolated from printing and dyeing sewage, such as the *S. putrefaciens* strain AS96, has a strong ability to treat dye-containing industrial effluents containing high concentrations of salt (Khalid et al., 2008). In addition, it can also affect some fish species as an opportunistic pathogen.

**Figure 1:**
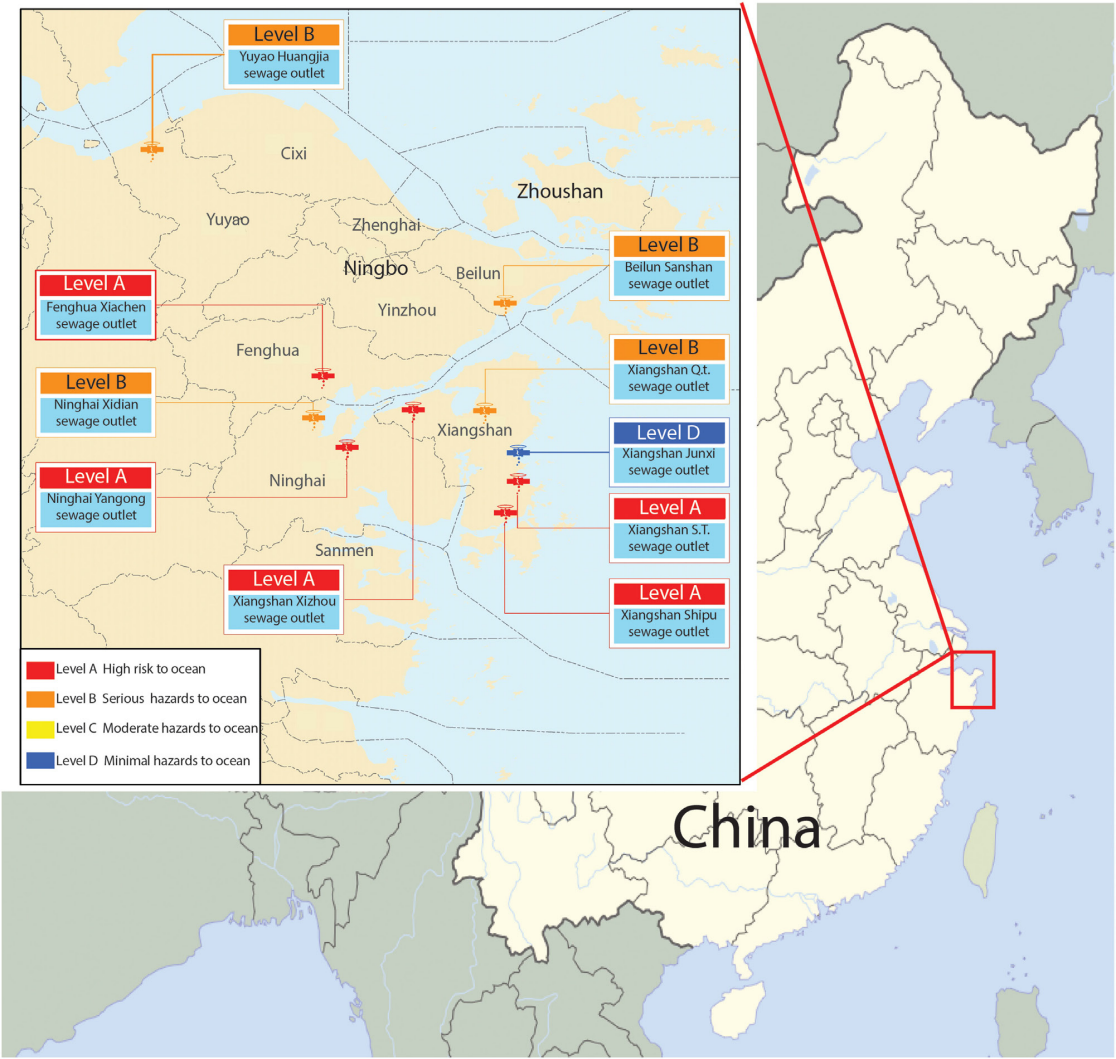
**Map of 10 terrestrial sewage outlets sites in coastal areas of Ningbo**.

In our previous research, we collected samples from 10 terrestrial sewage outlets in coastal areas of Ningbo (Figure 1; Figure S1 in Supplementary Material) and detected *S. putrefaciens* by 454 sequencing in March, May, August, and October, respectively (Weina, 2015). We found that *S. putrefaciens* was present in high abundance at almost every sewage outlet most of the time (Figure S2 in Supplementary Material). Xiangshan’s Qiangtou Comprehensive Sewage Outlet and the industrial sewage outlet at Shipu Aquatic Product Processing Park are among the top 10 sewage outlets in the coastal areas of Ningbo. Such an explosive proliferation might be related to the quorum-sensing function of bacteria (Goryachev et al., 2006). *S. putrefaciens* are mostly freshwater bacteria, and its colonization in the seawater near the terrestrial sewage outlets might affect the safety of aquatic products. Thus, it is significant and necessary to understand how *S. putrefaciens* colonizes the terrestrial sewage outlets in order to improve treatment of the contaminated river in contaminated water.

In this study, we employed differential proteomics, metabolomics, and real-time fluorescent quantitative PCR techniques to investigate the selective growth mechanism of *S. putrefaciens* and compare the results of growth in freshwater and sewage (Figure S3 in Supplementary Material). We also investigated the carbohydrate metabolic pathway to understand *S. putrefaciens* colonization in sewage.

## Materials and Methods

### Materials

*Shewanella putrefaciens* was isolated from sewage outlets and preserved at our laboratory (Figure S4 in Supplementary Material). Sewage wastewater was collected from the Xiangshan sewage outlet, with compositions as detailed in Table S1 in Supplementary Material. The total solids (TS) content of the sewage waste water was 3.1 ± 0.03 g/L and total chemical oxygen demand (TCOD) was 1241.5 ± 59.4 mg/L. All other reagents were purchased from TaKaRa Biotechnology (Dalian) Co., Ltd. and BIO-RAD (USA).

### Bacterial Culture

*Shewanella putrefaciens* isolated from the sewage outlets was inoculated in sterilized culture medium [based on Zhang et al. (2015)] in a thermostatic incubator for 24 h at 28°C. The bacterial culture was aliquoted and stored in 20% glycerol at −80°C under aseptic condition. The remaining bacteria culture was stored at 4°C. *S. putrefaciens* was grown in freshwater (W) as the control group (peptone 1%, beef extract 0.5%, NaCl 0.5%, deionized water) and sewage water (D) as the experiment group (peptone 1%, beef extract 0.5%). For both growth media, triplicate culture *of S. putrefaciens* was grown stationary in culture dished in a thermostatic incubator at 28°C for 24 h.

### Preparation of Membrane Protein Sample

The bacterial culture was centrifuged at 6000 rpm and 4°C for 10 min. The following preparation of membrane protein procedures was based on a method that we used previously (Weina, 2015; Zhang et al., 2015).

### Protein Purification

A 12% acrylamide gel was prepared; loading amount was 20 μL, containing the volume ratio of protein sample and 5× protein loading buffer (4:1). Before gel loading, the protein sample was heated to 100°C for 5 min in a water bath.

The loading volume was determined as 150 μL containing 200–250 μg total protein, and protein was diluted with hydration buffer to 150 μL (Zhang et al., 2015). The loading volume was calculated according to the results of protein qualitation. The strips were taken out from the refrigerator at −20°C and placed at room temperature for 1 h. The processed samples were put into the swelling tank, which was covered by the strips with the addition of mineral oil. Isoelectric focusing (IEF) was performed in PROTEAN Isoelectric Focusing System (Bio-Rad, USA). The temperatures of hydration and IEF were both set at 20°C. The hydration time was 12 h. The procedures of IEF are detailed in Table 1. Then, vertical SDS-PAGE electrophoresis was performed. After staining with Coomassie brilliant blue R-250 for 20 min, appropriate amount of decoloration solution was added (absolute alcohol:glacial acetic acid:water = 1:1:8). Decoloration was performed on a silent shaker, and a GS-800 Calibrated Densitometer was used for scanning. The PDQuest 8.0 software (Bio-Rad, USA) was used to identify proteins that were expressed differentially.

**Table 1:** **The protocol isoelectric focusing of protein samples**.

Phase	Voltage (V)	Duration (h)
1	500	1
2	1000	1
3	2000	3
4	500	1

**Phase**	**Balance buffer**	**Duration (h)**

5	Urea 6M, SDS 2% (W/V), 1.5M pH 8.8 Tris–HCl 25% (V/V), glycerine 20% (V/V), DTT 0.13M	0.25
6	Urea 6M, SDS 2% (W/V), 1.5M pH 8.8 Tris–HCl 25% (V/V), glycerine 20% (V/V), iodoacetamide 0.135M	0.25

### Protein Quantification

The non-interference protein assay kit SK3071 (Sangon Biotech, Shanghai) was used to quantify bacterial proteins using bovine serum protein as standards. Protein samples were quantified at a wavelength of 480 nm.

The differentially expressed proteins identified by PDQuest 8.0 were isolated from the gel and placed into clean 1.5 mL Eppendorf tubes. Samples were washed twice with MilliQ water, decolored with 50% alcohol, and digested with 20-ng pancreatic enzyme per microliter. Mass spectrometry was performed by using Autoflex speed™ MALDI-TOF-TOF (Bruker Dalton). The conditions were as follows: UV wavelength 355 nm, repetition rate 200 Hz, accelerating voltage 20,000 V, optimal mass resolution 1500 Da, and mass range 700–3200 Da. The signals were collected. The baseline peaks were filtered out, and the signal peaks were identified by the flexAnalysis (Bruker Dalton) software. The NCBI database was searched using BioTools (Bruker Dalton) to look for the matched proteins. The functions of the proteins were also queried (Sun et al., 2015).

### Transcription Level

The 0.1 g of bacteria was weighed and ground to powder in liquid nitrogen. Total RNA was extracted using 1-mL Trizol followed by a M-MuLV first strand cDNA synthesis kit (Sangon Biotech, Shanghai) to reverse transcribe RNA. Total RNA and cDNA concentration were measured by Nano Drop 2000 (Thermo Scientific, MA, USA). The primers and cDNA were identified by 2% agarose gel electrophoresis, and their integrity was evaluated.

The amino acid sequences of the differentially expressed proteins (detected by 2-DE) were downloaded from NCBI GenBank. After sequence alignment (Huson et al., 2007), the highly conserved regions were selected for primer design for quantitative PCR. Primers were designed using the Primer 5 software. Primer sequences are listed in Table S2 in Supplementary Material. Gene expressions were detected *via* SYBR green quantitative PCR of 20 μL reactions with 2 μL cDNA, 0.8 μL forward and reverse primers, 10 μL SYBR II, and 6.4 μL molecular grade water. The thermocycler was set to: 92°C 5 min; 92°C 5 s, 59°C 20 s, 40 cycles. Gene expressions were calculated by 2^−∆∆CT^ method, with 16s rRNA as the internal reference.

### Metabolomics

Bacterial pellets were fully grounded in liquid nitrogen. Cells were then dissolved by adding 5–10 mL methanol–water (2:1) solution and packaged into 1.5 mL centrifuge tubes. The mouth of each tube was covered by thin film, which was pierced by a syringe to make small holes. The tube was placed in the diaphragm vacuum pump. The extraction was performed with organic solvent until the samples could be frozen. The samples were combined, with triplicates for each group. NMR detection was performed after freezing and drying.

The samples were treated by Millipore filter membrane (Millipore Amicon^®^ ULTRA 3 kDa) and ACDSS reagent (DSS standard solution 5 mM). Then, NMR spectrometer (Bruker AV III 600 MHz spectrometer, equipped with a reverse probe) was used for mass spectrometry. The specific parameters were as follows: temperature 298 K, NMR frequency 600.13 MHz, sampling times 128, sample delay 1, frequency domain 65,536, spectral width 12,019.231, number of sampling points 32,768, and pulse sequence noesypr1d/noesygppr1d. Data were analyzed using the Chenomx NMR suit software (version 7.6, Canada).

### Statistical Analysis

Differential expressions at the transcriptional level used protein score. Protein score is −10 × log(*P*), where *P* is the probability that the observed match is a random event. Protein scores greater than 56 are significant (*P* < 0.05). NMR data were analyzed using the Chenomx NMR suit software (version 7.6, Canada).

## Results

### Quality Control of Membrane Proteins

**Figure 2:**
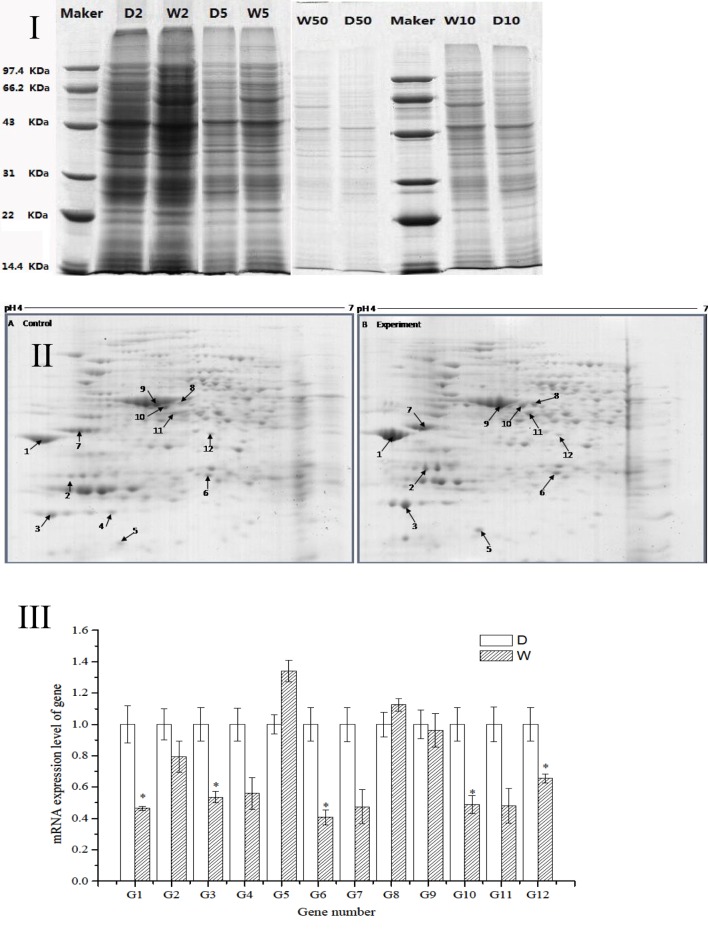
**(I)** SDS-PAGE electropherogram. D, fresh water; W, sewage. **(II)** The 2D-PAGE protein map of *S. putrefaciens* under the two different environment. A, Fresh water; B, sewage. Arrows and spot numbers refer to differentially expressed proteins. **(III)** The RT-qPCR of differential expression gene of *S. putrefaciens*. D, fresh water; W, sewage.

The regression equation *y* = −0.005*x* + 0.481 (*R*^2^ = 0.998) was obtained from the protein assay kit. The total protein concentrations of *S. putrefaciens* grown in sewage (D) and freshwater (W) were determined as 8.46 and 9.74 μg/μL, respectively. The protein samples were serial diluted to 2-, 5-, 10-, and 50-fold. SDS-PAGE electrophoresis showed clear strips for 10-fold dilution. The corresponding protein concentration was approximately 0.8–1.0 μg/μL, which was the loading concentration of 2-DE sample (Figure 2I).

### Differential Expressions of Proteins

The proteomic spectra obtained by two-dimensional gel electrophoresis is shown in Figure 2II. Analysis using the PDQust8.0 software identified 15 proteins that were differentially expressed between the control and experimental groups. MALDI-TOF-MS-MS was performed, and 12 differentially expressed proteins were identified: outer membrane protein Omp35, outer membrane protein W (OmpW), deoxyriboaldolase, superoxide dismutase (SOD), β-ketoacyl synthase, 2-oxoglutarate dehydrogenase, succinyl coenzyme A synthetase β subunit, and deoxyribose-phosphate pyrophosphokinase (Table 2). Comparing *S. putrefaciens* growth in freshwater and sewage medium, 11 proteins were upregulated expression and 1 protein (SOD) was downregulated. Eight of these proteins were increased by more than 50% in wastewater. Deoxyribose-phosphate aldolase (P2) showed the largest increase, with approximately fivefold increase in expression, followed by outer membrane protein OmpW (P3, 3.67×), outer membrane porin Omp35 (P1, 3.28×), and beta-ketoacyl synthase (P8, 3.18×). KEGG analysis of these proteins show that they are involved in multiple functions, including the formation of biological membranes (Omp35, OmpW), energy metabolism (SOD, deoxyribose-phosphate pyrophosphokinase), fatty acid metabolism (beta-ketoacyl synthase), secondary metabolism, TCA cycle, lysine degradation (2-oxoglutarate reductase), and propionic acid metabolism (succinyl coenzyme A synthetase).

**Table 2:** **The mass spectrometry result of different proteins from *S. putrefaciens* in freshwater and sewage**.

No.	Accession number	Protein name	Matched peptides	Mascot score	Theoretical MW (kDa)/PI	Threshold (*P* < 0.05)	Species
1	gi|386312552	Outer membrane porin, Omp35	13	148	37,959/4.64	56	*S. putrefaciens* 200
2	gi|146293916	Deoxyribose-phosphate aldolase	8	92	27,568/4.75	56	*S. putrefaciens* CN-32
3	gi|386313173	Outer membrane protein, OmpW	3	96	22,968/4.76	56	*S. putrefaciens* 200
4	gi|146292787	Superoxide dismutase	6	68	21,613/4.94	56	*S. putrefaciens* CN-32
5	gi|146292406	Hypothetical protein Sputcn32_1303	7	140	19,533/5.37	56	*S. putrefaciens* CN-32
6	gi|146294960	Extracellular solute-binding protein	12	134	29,511/6.15	56	*S. putrefaciens* CN-32
7	gi|146292153	OmpA/MotB domain-containing protein	7	81	40,022/4.82	56	*S. putrefaciens* CN-32
8	gi|146293541	Beta-ketoacyl synthase	8	101	44,387/5.29	56	*S. putrefaciens* CN-32
9	gi|146294855	Elongation factor Tu	15	299	43,547/5.08	56	*S. putrefaciens* CN-32
10	gi|146293364	Dihydrolipoamide succinyltransferase	5	60	43,234/5.34	56	*S. putrefaciens* CN-32
11	gi|146293363	Succinyl-CoA synthetase subunit beta	9	166	41,699/5.32	56	*S. putrefaciens* CN-32
12	gi|146291902	Ribose-phosphate pyrophosphokinase	4	63	34,249/5.46	56	*S. putrefaciens* CN-32

### Differential Expressions at the Transcriptional Level

The sequences of the above 12 differentially expressed proteins were aligned with sequences downloaded from NCBI [Database: putrefaciens (22,390 sequences; 7,423,079 residues)]. The specific primers were designed and synthesized (Table S2 in Supplementary Material) for qRT-PCR. As shown in Figure 2III, 2 genes from *S. putrefaciens* were upregulated under sewage stress (P5: hypothetical protein; P8: beta-ketoacyl synthase), and 10 genes were downregulated. The expressions of G1, G3, G6, G10, and G12 in the experimental group showed significant differences compared to those in the control group (*P* < 0.05). As seen from the results of 2-DE and RT-qPCR, the upregulated expressions of Omp35, deoxyribose-phosphate aldolase, and OmpW in proteomics analysis were not verified by RT-qPCR. We believe that the expression on the protein level may be inconsistent with the expression at the mRNA level. There are many reports that have reached similar conclusions. For instance, Evguenieva-Hackenberg and Klug (2011) believed that in bacteria and archaebacteria, genomics results revealed the important role of posttranscriptional regulation in protein synthesis, as well as the low correlation between the expression on protein level and mRNA level.

### Metabolomics Analysis

**Figure 3:**
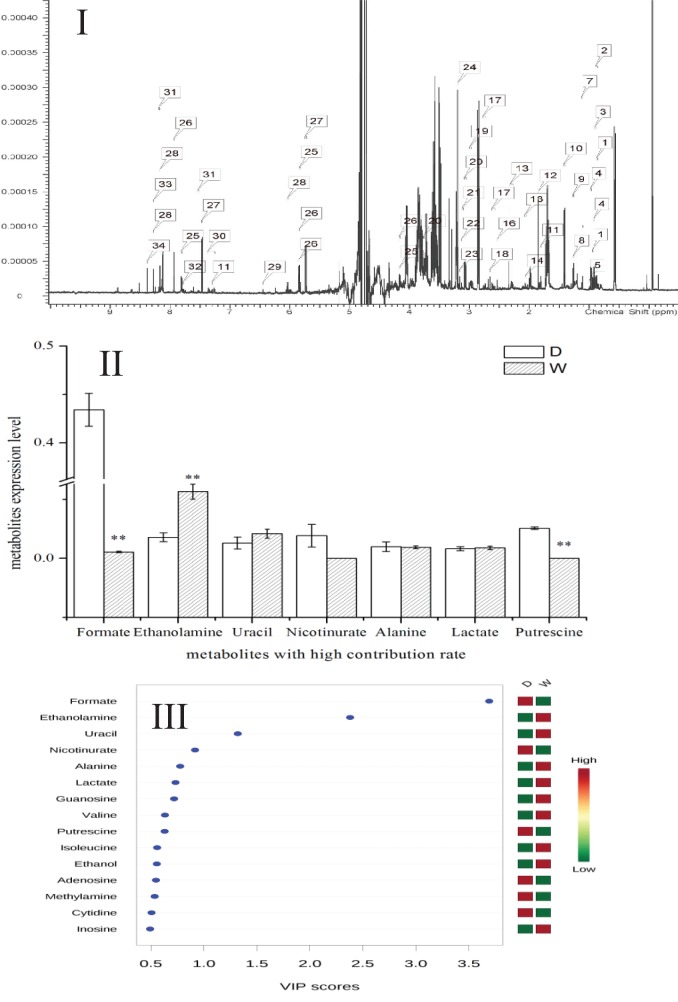
**(I)** NMR ownership figure of metabolites from *S. putrefaciens*. **(II)** Expression levels of bacteria in freshwater and water environment of high contribution rate metabolites. W, water group (control); D, sewage group (experimental) (**P < 0.01). **(III)** The PLS analysis graphics of intracellular metabolites from *S. putrefaciens*. VIP, variable importance in projection.

Metabolomics is a new tool to analyze the variations in metabolites with small molecular mass after stimulation or disturbance in a biological system using qualitative and quantitative methods. With metabolomics, a biological system can be analyzed dynamically (Nicholson et al., 1999). The intracellular metabolites of *S. putrefaciens* grown in sewage and freshwater environment were extracted for NMR analysis. Twenty-seven differentially metabolized products were detected (Figure 3I; Table 3), including alcohols (ethanol, isopropanol), amines (dimethylamine, ethanolamine), amino acids (alanine, leucine), amine compounds (bilinerurine), nucleic acid compounds (nucleosides, inosines), and organic acids (formate, acetate). The expression levels in bacteria in sewage and freshwater were analyzed by partial least squares (PLS). The results showed that formate, ethanolamine, uracil, acetate, and putrescine scored highly. They contribute greatly to the difference between the two types of growth media. Of note, the score of formate was far higher than that of other products. The concentrations showed a highly significant difference in the two environments (Figures 3II,III). In sewage, the concentrations of formate and putrescine were decreased. On the contrary, the concentration of ethanolamine increased. These metabolites are related to the decomposition of aromatic hydrocarbons and alkanes, metabolism of pyruvic acid, and the synthesis of secondary metabolites. Compared to the freshwater environment, the intracellular metabolite of *S. putrefaciens* grown in sewage is more complex in composition, with an increase in multiple metabolites. From this, it is inferred that the metabolic pathways related to these metabolites are also upregulated.

**Table 3:** **Intracellular metabolites information table of *S. putrefaciens***.

		Concentration (%)			
Category	Name	Fresh water	Sewage	Ratio^a^	NMR number
Alcohols	Ethanol	0.0098	0.0091	0.93	7
	Isopropanol	0.0007	0	0*	6
Amines	Dimethylamine	0.0016	0.0019	1.23	18
	Ethanolamine	0.018	0.056	3.15**	20
	Methylamine	0.0075	0	0	16
	Putrescine	0.017	0	0**	19
Amino acids	Alanine	0.0099	0.0094	0.95	10
	Aspartate	0	0	–	17
	Betaine	0.00067	0	0*	24
	Glutamate	0	0	–	13
	Isoleucine	0.0013	0.0032	2.46*	1
	Leucine	0.0012	0	0	3
	Methionine	0.0016	0	0	14
	Phenylalanine	0	0	–	30
	Threonine	0.0012	0.0022	1.81	8
	Valine	0.0018	0.0042	2.33**	4
	2-Alanine	0	0	–	21
Ammoniums compounds	Choline	0.00063	0.0007	1.11	22
	sn-Glycero-3-phosphocholine	0	0	–	23
Nucleic acid components	Adenosine	0.0070	0	0**	33
	Cytidine	0.0060	0	0	32
	Guanosine	0.0075	0.0074	0.99	26
	Inosine	0.0044	0.0040	0.92	28
	Thymine	0	0	–	11
	Uracil	0.013	0.021	1.60	27
	Uridine	0.00053	0	0	25
Organic acids	2-Hydroxybutyrate	0.0053	0	0**	2
	Acetate	0.024	0.0080	0.33	12
	Formate	0.33	0.0054	0.02**	34
	Fumarate	0	0	–	29
	Isobutyrate	0.0008	0	0	5
	Lactate	0.0082	0.0090	1.09	9
	Succinate	0.0021	0.0017	0.80	15
Others	Nicotinurate	0.0192	0	0	31

*^a^The intracellular metabolites concentration of sewage samples versus control samples. >1, upregulation; <1, downregulation; –, no detect*.

*0.01 < P < 0.05.

**P < 0.01.

## Discussion

**Figure 4:**
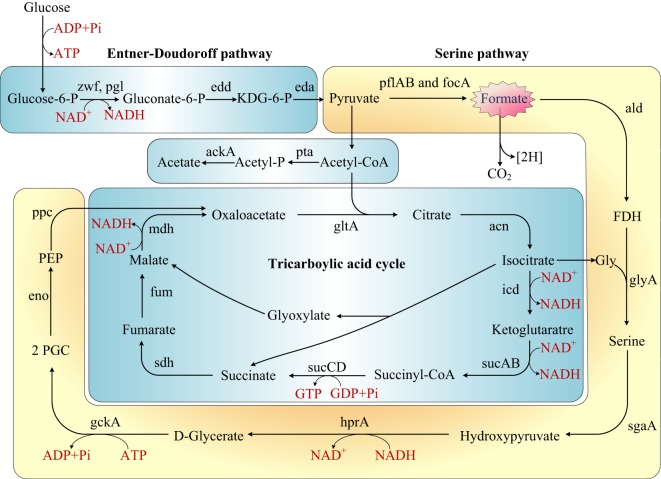
**Rewiring of transcriptional regulatory networks in the carbohydrate mechanisms of *Shewanella* which colonized in the sewage according to KEGG and previous research (Scott and Nealson, 1994; Fredrickson et al., 2008; Pinchuk et al., 2011)**. Genes that encode the enzymes in this schematic are referred to by their commonly used names. KDG-6-P, 2-keto-3-deoxy-6-phosphogluconate; P, phosphate; FDH, formaldehyde; 2PGC, 2 phosphoglycerate; Gly, glycine; PEP, phosphoenolpyruvate.

The first published description of *Shewanella* in 1988 reported the capability of manganese and iron oxides metabolism (Myers and Nealson, 1988). Later studies on *Shewanella* focused mainly on three characteristics of the bacterium: (1) the electrical conductivity (Lower et al., 2001; Heidelberg et al., 2002; Kim et al., 2002; Liu et al., 2004; Gorby et al., 2006), (2) iron reduction (Beliaev and Saffarini, 1998; Kim et al., 1999), and (3) anaerobic regulation in different *Shewanella* species (Ding et al., 2016; White et al., 2016). Some researchers also reported the ability of *Shewanella* to treat azo dye waste water and analyzed the mechanism byproducts (Pearce et al., 2006; Khalid et al., 2008; Liu et al., 2013; Yu et al., 2015). Our research is the first to report the colonization of *S. putrefaciens* in the terrestrial sewage outlets by analysis of protein and gene expression and metabolic products. Our results illustrated the capability of *S. putrefaciens* to grow under sewage conditions (Figure S5 in Supplementary Material). The authors also propose the carbohydrate mechanism for the colonization of sewage by *S. putrefaciens* (Figure 4).

### Diverse Metabolic Products Acclimate *S. putrefaciens* to Contaminated Environments

Formate is an important metabolite in many aerobic bacteria, anaerobic bacteria, and some yeasts. In our study, the concentration of formate decreased in sewage. We proposed that *S. putrefaciens* undergoes formate metabolism under wastewater stress (Figure 4). Before the genome of *Shewanella* was sequenced, it was thought to use a restricted range of substrates as carbon and energy sources (formate, lactate, and pyruvate). However, the genome annotation demonstrated that *Shewanella* can use a range of amino acids, carboxylates, sugars, and nucleosides as carbon and energy sources (Fredrickson et al., 2008). *S. putrefaciens* has been previously proposed to dissimilate glucose *via* the Entner–Doudoroff pathway (Scott and Nealson, 1994; Fredrickson et al., 2008). The TCA cycle is a principal route of carbon metabolism during aerobic growth. However, the TCA cycle is truncated and no longer plays an important role in anaerobic metabolism. In an anaerobic environment, *S. putrefaciens* grows with formate as the sole source of carbon and energy (Scott and Nealson, 1994). *S. putrefaciens* is the first member of the gamma-purple family of Proteobacteria known to utilize the serine pathway, which plays an important role in *S. putrefaciens*’s ability to adapt to anaerobic environments. We suggest that under freshwater stress, *S. putrefaciens* switches to the anaerobic pathway (serine pathway, Figure 4) to produce diverse metabolic products to acclimate to contaminated environments.

By serving as the auxiliary reducer in complex substrates, formate is an intermediate in the energy-saving pathway in complex environments (Ferry, 1990). As the substrate for formate dehydrogenase (FDH), formate is oxidized to CO_2_ and NADH in the presence of FDH and NAD^+^ (Schütte et al., 1976; Popov and Lamzin, 1994). FDH is a d-2-hydroxyaciddehydrogenase consisting of two identical subunits, but no metal ions or prosthetic groups. It showed high specificity to formic acid and NAD^+^ and extreme sensitivity to O_2_ as a NAD^+^-dependent oxidase. According to the KEGG pathway, FDH is associated with the two-component signal transduction system (TCS), which is present in most bacteria for signal transduction. Consisting of histidine protein kinases and response mediator proteins, FDH senses the external signals and quickly transmits the signals to the bacteria. As a consequence, the transcription, translation, and expression of a series of genes and the modification of the expression products are initiated (Stock et al., 2000; Galperin et al., 2001). Thus, the adaptation of the bacteria to the external environment is improved.

We infer that in sewage environments, *S. putrefaciens* upregulates the TCS mechanism in response to stress. With the upregulation of formaldehyde (FDH), formate is oxidized to produce more NADH, which provides more energy to the bacteria growing under sewage stress and reduces the harm brought by toxic substances. The energy is supplied for the bacteria to enable growth in the sewage outlets.

### Effects of Sewage on Expressions of Proteins of *S. putrefaciens*

In our research, 11 proteins were involved in the formation of biological membranes, energy metabolism, fatty acid metabolism, secondary metabolism, TCA cycle, lysine degradation, and propionic acid metabolism. Succinyl coenzyme A synthetase, an important enzyme in the TCA cycle, was upregulated when cultured in the sewage (Figure 4).

In this study, elongation factor Tu (EF-Tu) expression within sewage wastewater environments was identified as having an obvious upregulated expression pattern, which indicates the need for vigorous synthesis of relative proteins against wastewater. Ramiah et al. (2008) reported that the upregulated EF-Tu played some role in *Lactobacillus plantarum* 423 adherences to Caco-2 cells and may be regarded as a multifunctional protein that assisted *L. plantarum* 423 in adapting to environmental changes. Ristic et al. (2008) showed that the accumulation of chloroplast EF-Tu (upregulation), induced by heat stress, conferred heat tolerance by acting as a molecular chaperone and protecting chloroplast proteins from thermal aggregation and inactivation. This suggests that the expression of EF-Tu, whether gene or protein, was upregulated in *S. putrefaciens* extracted from sewage wastewater.

The other significant upregulated expression protein was the outer membrane in the sewage culture (Omp35, OmpW, etc). The outer membrane is located outside the cytoplasmic membrane (inner membrane) and peptidoglycan, encompassing the entire bacterium. It is a unique component for the cell wall of Gram-negative bacteria. Outer membrane proteins are the main component in the outer membrane of Gram-negative bacteria. They play an important role not only in substance transport, morphology maintenance, and synthesis of relevant substances but also in the adaptation of the organisms to the external environment (Lin et al., 2002). Omp35 is a 35-kDa outer membrane protein, which participates in the electron transport on the respiratory chain in the environment of fumaric acid esters, nitrates, and iron (III) (Maier and Myers, 2004). This protein is necessary for the normal growth of bacteria in such environments. Another protein, OmpW, is a type of micropore protein, belonging to the family of small outer membrane proteins. It consists of 200–230 amino acids, which can form a barrel-shaped channel composed of eight β-folds to assist the passage of hydrophobic molecules through the outer membrane of the bacteria (Hong et al., 2006). OmpW is present in a variety of bacteria, but its biological functions are not yet elucidated. Existing research suggests that this outer membrane protein may be associated with the permeation, oxidation, acquisition of temperature and nutrients, and drug resistance (Nikaido, 2003). In addition, the upregulation of OmpW is also correlated with an increase of bacterial virulence (Wu et al., 2013).

It has been demonstrated that the outer membrane proteins play an important role in the formation of biofilm. The expressions of OmpW and Omp35 were both upregulated in sewage. We infer that these two outer membrane proteins may be associated with the formation of biofilm by providing the structural and material basis and safety for the colonization of bacteria in sewage. The chemical oxygen demand (COD), total phosphorus (TP), and total nitrogen (TN) are important indicators for the degree of organic matter pollution of water. The 2011 monitoring results of terrestrial sewage outlets into the sea in Ningbo City showed that COD, TP, and TN of Xiangshan’s Qiangtou comprehensive Sewage Outlet severely exceeded the standards. Water pollution reduces the bacterial diversity and induces the enrichment of the microbial community that can decompose the pollutants.

## Author Contributions

XS proposed the study and provided guidance to all co-authors. JX and WH designed and conducted the experiment, collected samples, and determined analyte concentrations. WH, DZ, and ZW analyzed bioreactor system data and conducted calculations. JZ, JS, and YL performed all methods to separate the microbial community, including corresponding data analysis and figure preparation. JX prepared other figures. XS, JX, and WH wrote the manuscript, with revisions from all the other authors.

## Conflict of Interest Statement

The authors declare that the research was conducted in the absence of any commercial or financial relationships that could be construed as a potential conflict of interest.
