# Supplementary material for: A Comparison of Molecular Biology Mechanism of Shewanella putrefaciens between Fresh and Terrestrial Sewage Wastewater
Source: Front Bioeng Biotechnol. 2016 Nov 4;4:86. doi: 10.3389/fbioe.2016.00086 (PMC5095135; doi:10.3389/fbioe.2016.00086)
Supplement: Supplementary file 1 [file Presentation_1.pdf]

A comparison of molecular biology mechanism of *Shewanella putrefaciens* between fresh and terrestrial sewage wastewater

Jiajie Xu[1,2], Weina He[1], Zhonghua Wang[1], Dijun Zhang[1], Jing Sun[1], Jun Zhou[1], Yanyan Li[1,3], Xiurong Su[1]

[1]School of Marine Science, Ningbo University, Zhejiang, China, [2]College of Engineering, China Agricultural University, Beijing, China, [3]Department of Food Science, Cornell University, Ithaca, NY 14853, USA

Correspondence to: suxiurong@nbu.edu.cn

Supplementary Information:

Tables S1-S2

Figures S1-S5

Table S1 The compositions of the waste water in Xiangshan sewage outlet

	TS (g L^{-1}) (n=6)	Ammonia (mg L^{-1}) (n=6)	TCOD (mg L^{-1}) (n=6)	BOD (mg L^{-1}) (n=6)	pH (n=6)
Waste water	3.1±0.03	23.68±1.40	1241.5±59.4	315.6±18.7	6.7±0.5

Table S2 The primers used in the qRT-PCR experiment

Protein number	Primer	Sequence(5'-3')	Product size
P1	forward	ACCGTTTAGTGAGCGCTCAA	259bp
	reverse	AACTTAGCCTGAGCCACACC	
P2	forward	TGGAGACACCCAATCAGCAC	238bp
	reverse	GCTTATTCGCCGTGCTTCTG	
P3	forward	ATTGGTGCAGGCGTGAACTA	124bp
	reverse	AATCTAAGCCAACCTGCGCT	
P4	forward	AGTGCTGTCAGTCAGTGGTG	170bp
	reverse	CAGATGCCATCAATGCAGCC	
P5	forward	TGCTGTTTCTGTCGGAGGTC	222bp
	reverse	TCGAAGGTCAAATCTCGGGC	
P6	forward	TTAAGCTCGCCACCACAACA	168bp
	reverse	GGGCGTGGGTCATCACTAAA	
P7	forward	TTGTCTGAGTCGACAGGTGC	104bp
	reverse	AGCAACTACTGGGGCCTAGA	
P8	forward	GACGCGCCATAACCAATCAC	179bp
	reverse	ATGGGCGCACTGTCAACTAA	
P9	forward	ACTGTGGACGGTAGCCTTTG	190bp
	reverse	GAAGGTCGTGCAGGTGAGAA	
P10	forward	TACGACCGCCTACACCTGTA	221bp

	reverse	CCAAGAGGGCGATACCGTAC	
P11	forward	AACGCTTCAGCAACACGTTC	250bp
	reverse	ATGGTAACGCACTGTTCCGT	
P12	forward	CGCCAGATCGGAATCATCGA	267bp
	reverse	CGTAGCGCACGTGTACCTAT	

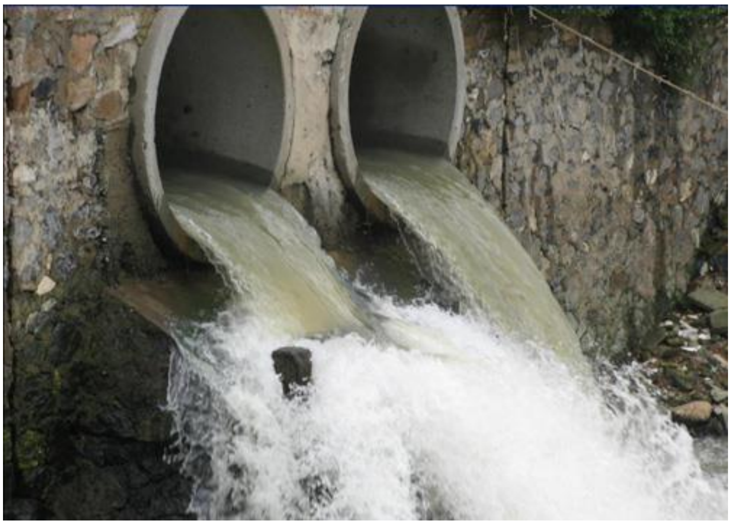

Fig. S1 Photographs of terrestrial sewage outlets

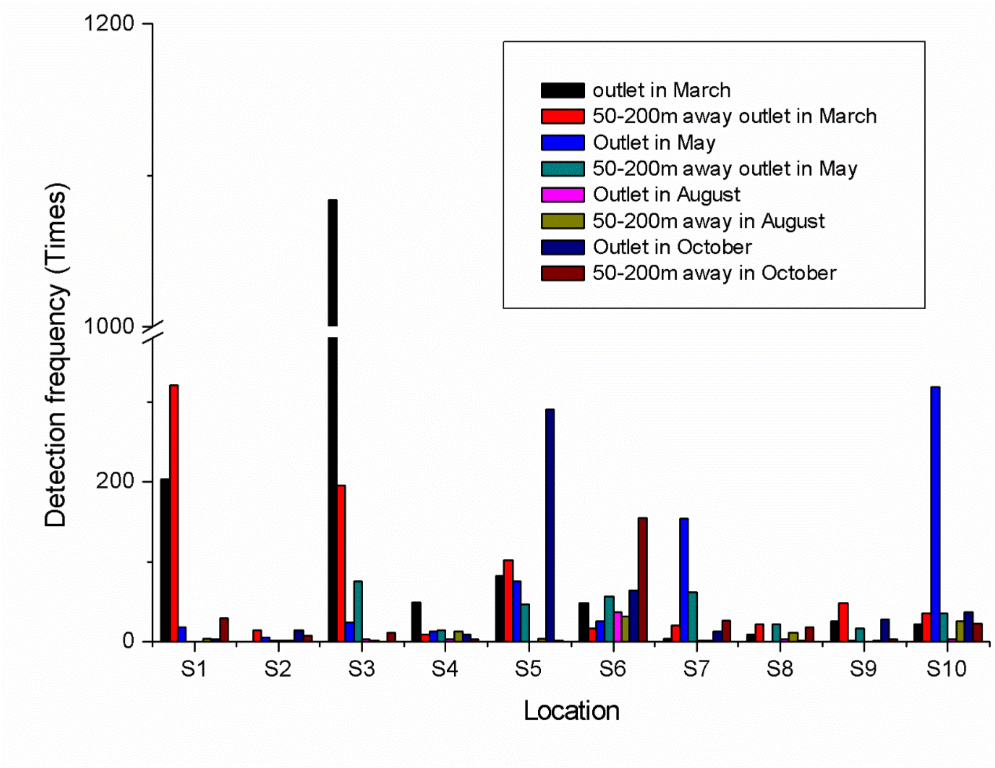

Fig. S2 The detection frequency of *S. putrefaciens* in different 10 terrestrial sewage outlets in coastal areas of Ningbo. S1: Xiangshan Junxi sewage outlet; S2: Xiangshan S.T. sewage outlet; S3: Xiangshan Shipu sewage outlet; S4: Xiangshan Xizhou sewage outlet; S5: Xiangshan Q.T. sewage outlet; S6: Beilun Sanshan sewage outlet; S7: Ninghai Xidian sewage outlet; S8: Fenghua Xiachen sewage outlet; S9: Ninghai Yangong sewage outlet; S10: Yuyao Huangjiang sewage outlet.

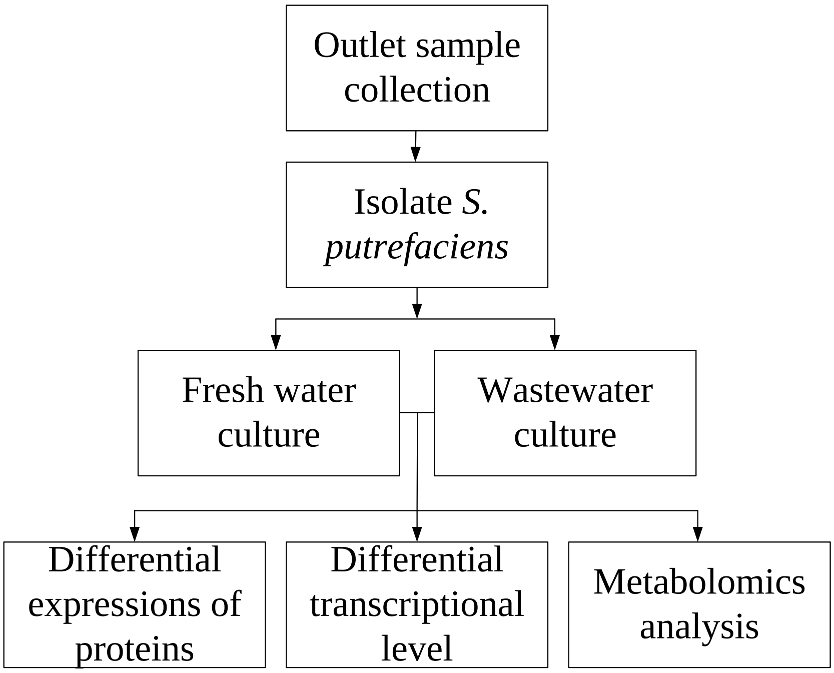

Fig. S3 A diagram of overview of *S putrefaciens* study

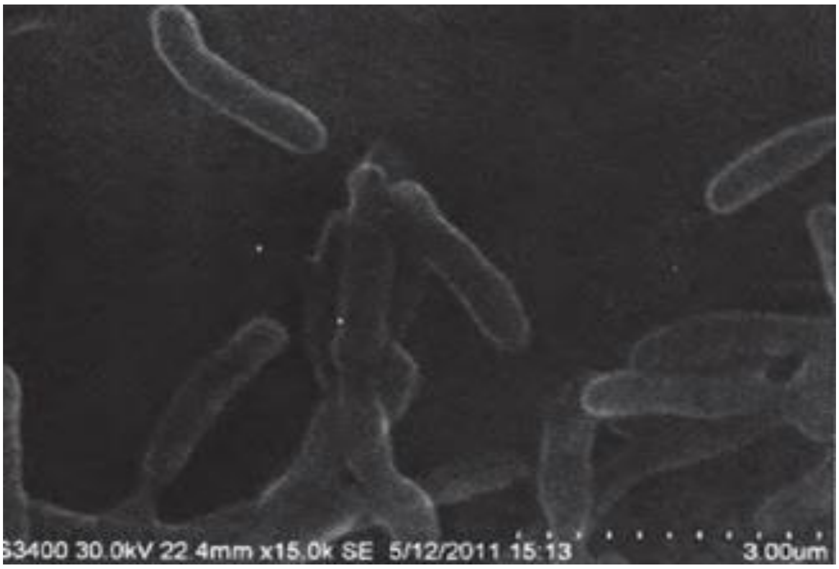

Fig. S4 photomicrograph of isolated *S. putrefaciens*

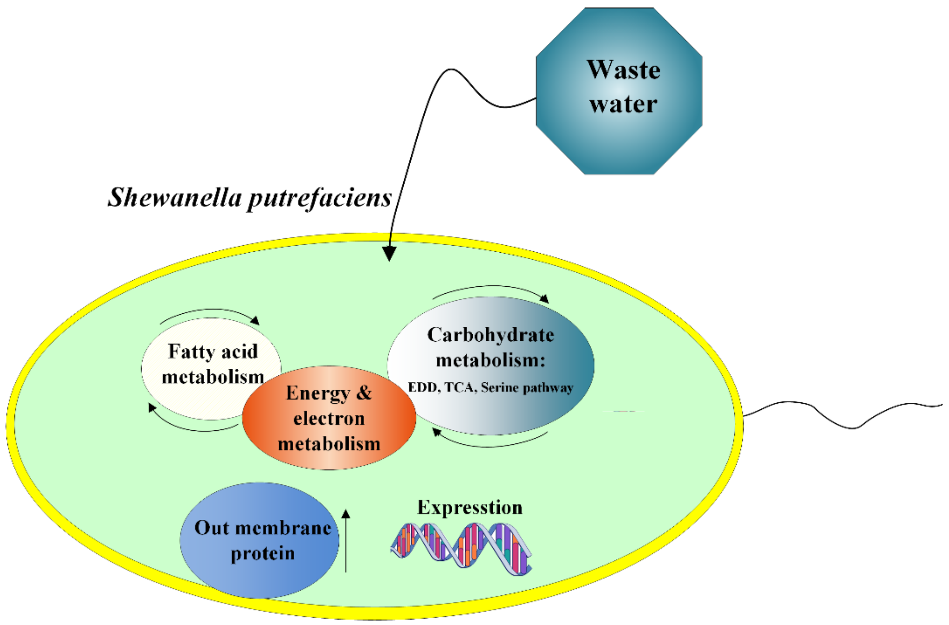

Fig. S5 Schematic representation of molecular biology mechanism in *S. putrefaciens* under sewage affection.
